# The use of Maytenus ilicifolia to prevent cisplatin-induced ototoxicity

**DOI:** 10.1016/S1808-8694(15)31381-1

**Published:** 2015-10-17

**Authors:** Cristiane Akemi Kasse, Oswaldo L M Cruz, Luis C N Iha, Henrique O Costa, Elaine C Lopes, Flávia Coelho

**Affiliations:** 1 Master’s and Doctoral degrees, Universidade Federal de São Paulo, UNIFESP-EPM. Research professor, Universidade Bandeirantes, UNIBAN; 2 Livre docente (habilitation) professor, Universidade de São Paulo, USP. Affiliated professor, Otorhinolaryngology discipline, Universidade Federal de São Paulo, UNIFESP; 3 Master’s degree, Universidade Federal de São Paulo, UNIFESP. Doctoral student, Universidade Federal de São Paulo, UNIFESP; 4 Doctoral degree, Santa Casa de Misericórdia de São Paulo. Professor, Santa Casa de Misericórdia de São Paulo; 5 Doctor in pharmacology, Universidade Federal de São Paulo, UNIFESP. Researcher, Instituto de Ciências Avançadas em Otorrinolaringologia, ICAO; 6 Veterinarian, Santa Casa de Misericórdia de São Paulo and the Hospital Sírio Libanês. Researcher, Instituto de Ciências Avançadas em Otorrinolaringologia, ICAO. Department of human communication disorders, Otorhinolaryngology discipline, Universidade Federal de São Paulo (UNIFESP)

**Keywords:** maytenus, free radicals, drug toxicity

## Abstract

*Maytenus ilicifolia* is a native plant from South America, with several medicinal properties including antioxidant effects.

**Aim:**

using an original cisplatin induced ototoxicity model, we evaluated a possible otoprotection caused by Maytenus ilicifolia extract.

**Materials and methods:**

clinical and experimental study design with female albino guinea pigs divided in groups as follows: 9 animals receiving cisplatin only (three doses of 7.5mg/kg/day), 4 animals receiving the plant extract only, 10 animals receiving the cisplatin protocol and 1g/kg/day of extract for 8 days, 5 animals with cisplatin and 3g/kg/day of extract for 8 days, and 5 animals receiving extract for 3 weeks and cisplatin in the last week. The tests were distortion product otoacoustic emissions, brainstem auditory response, before and after medication and scanning electron microscopy.

**Results:**

the animals receiving cisplatin plus plant extract, had alterations in all the tests, showing lesions on the basal cochlear region under electron microscopy.

**Conclusions:**

Despite of the plant extract’s antioxidant effect, it was not sufficient to protect the cochlea against cisplatin ototoxicity.

## INTRODUCTION

A major challenge for Otology in this century has been the treatment of sensorial dysacusis; this condition has become more frequent due to noise exposure, aging and use of medication, among other causes. Recent studies of medicinal plants^1^, ^2^, ^3^ have attracted the attention of our group in finding otoprotecting properties to attenuate or avoid cochlear injury.

Maytenus ilicifolia is a native plant in many regions of South America, including southeast Brazil. This plant has various possible medicinal actions including analgesic, anti-inflammatory,^1^ antitumor, antiulcer^2^ and antioxidanting3 effects. We chose Maytenus ilicifolia to investigate its possible otoprotective effects because it has antioxydating properties (presence of flavonoids and alkaloids),^4^, ^5^ few side effects in human beings, and minimizes the toxic action of a variety of agents.^6^, ^7^, ^8^

We chose cisplatin for producing cochlear injuries. This drug is an important chemotherapy agent for the treatment of solid tumors in adults and children; it may cause (often irreversible) auditory injury and is nephrotoxic,^9^ also causing significant gastrointestinal and neurological side effects.^10^, ^11^ Cisplatin injures various cochlear structures including hair cells, supporting cells, the vascular striae and the auditory nerve.^12^, ^13^ Cisplatin produces free radicals that affect the antioxidative cell system, leading to cell death.^14^ The degree of injury depends on the dose and administration route.^12^ We tested a few ototoxicity protocols in guinea pigs,^15^, ^16^, ^17^ but did not encounter the injury level described in some of these protocols,^17^ or there was a high mortality rate with ototoxic doses.^16^ For this reason we developed an original protocol which yielded injury in three cochlear gyri, ratified by otoacoustic emission distortion product tests (OAEDP), the brainstem auditory evoked potential (BAEP) and scanning electron microscopy (EM); this protocol had a lower lethality rate. The ideal dose was 22.5 mg/kg given intraperitoneally, divided into three doses, spaced five days between the first and second dose and one day between the last doses.^18^

The purpose of this study was to verify by function tests any possible otoprotective action provided by the aqueous extract of Maytenus ilicifolia in cisplatin-treated guinea pigs.

## METHOD

The Research Ethics Committee of our university approved this clinical and experimental study (number 1461/04). The animals were 3 to 4 month female albino guinea pigs weighing from 350 to 450g. The choice of sex, weight and lack of pigmentation was based on clinical observation and data in the literature^19^ on sensitivity, cisplatin ototoxicity, and increased animal survival and test response uniformity. All animals were healthy and had normal otoscopies.

### Obtaining the Aqueous Extract of Maytenus ilicifolia

The Centro de Pesquisas Químicas, Biológicas e Agrícolas provided the Maytenus ilicifolia extract. The species is cultivated in this center by doctors Pedro Mellilo Magalhães and Ílio Montanari Júnior under controlled soil fertility, humidity, sunlight, temperature, and herbivore and soil pollution agricultural conditions.

The Maytenus ilicifolia leaf powder was extracted by infusion (5%, 50 g/l) in water during 30 minutes at 73ºC. The resulting liquid was filtered, concentrated to 150 ml in a vacuum evaporator at 50ºC and freeze-dried to yield the aqueous extract of Maytenus ilicifolia.

### Assessment of Auditory Function

All animals underwent BAEP and OAEDP testing to measure auditory thresholds and the wholeness of the outer hair cells (OHCs). The test device was a Bio-logic, Navigator-Pro and Audix model. Guinea pigs were sedated with xylazine (10 mg/kg) and ketamine chloridrate (40 mg/kg).

The test environment was acoustically adequate and the electrodes were placed over the mastoid region of each ear and the vertex of the head; earphones were placed over the corresponding ears.

A monochannel click stimulus was used for BAEP testing (0.1 to 1.5 kHz filter, 10.66ms window, 100ms click duration, 13.3 times per second stimulus rate, 50,000 mean gain, visualized on an oscilloscope). Analyzed frequencies were 1 to 3 kHz. The threshold was defined as the lowest intensity that reproduced wave deflection tracings. Responses were given in SPL (sound pressure level).

We used the OAEDP as it is a highly sensitive test for cisplatin-provoked OHC injury.^15^, ^16^ Responses were analyzed at 500, 1, 2, 3, 4 and 8 kHz, with L1 and L2 from 65 to 55 SPL intensities respectively, and a 1.2 F1/F2 ratio. A probe for emitting sounds (f1 and f2) was placed over the ear that was being tested until a response was obtained. We compared the parameters background noise (NF) and the response (DP), and analyzed the difference (DP-NF) before and after the treatment.

Guinea pigs that had prior altered tests were excluded from the study.

### Scanning Electron Microscopy Investigation

Scanning electron microscopy was done on the animals to demonstrate the cisplatin-induced OHC injuries, particularly in the basal gyrus^16^ and to observe possible morphological changes in the animals treated with the aqueous extract.

At the end of treatment the animals were sacrificed using a lethal injection of the anesthetic (ketamine), then decapitated for microsurgical cochlear dissection.

The cochlear was fixated by the perfusion technique using a 2.5% glutaraldehyde solution, and buffered to pH 7.4 with acetate during 24 hours; a sufficient amount was injected into the round window until perilymph exited the apex. The material was then placed in a buffered sodium cacodilate 0.1 M, ph 7.2 solution during 12 hours. Ethanol dehydration was done increasing its concentration from 50% to 100%, in steps of 30 minutes. The critical point was done during 60 minutes, and a gold bath was done during one hour. The samples were placed in supports and fixated with enamel for visualization in a JEOL model 5300 EM device, at 1500 and 2000X magnifications. We observed the three OHC layers and one inner hair cell layer in three and a half gyri; we chose those areas with fewer artifacts and less tissue loss. Gyri were glued separately and the areas that were difficult to visualize were discarded.

### Treatment

The groups were divided as follows:

Group 1: 10 guinea pigs (20 ears) were given 3 doses of cisplatin (7.5 mg/kg/d) on the 1st, 5th and 6th days.
Group 2: 10 guinea pigs (20 ears) were given only the aqueous extract (3 g/kg/d) daily for 7 days.
Group 3: 10 guinea pigs (20 ears) were given the aqueous extract (1 g/kg/d) daily for 7 days and 3 doses of cisplatin.
Group 4: 5 guinea pigs (10 ears) were given the aqueous extract (3 g/kg/d) daily for 7 days and 3 doses of cisplatin.
Group 5: 5 guinea pigs (10 ears) were given the aqueous extract (1g/kg/d) for 3 weeks and 3 doses of cisplatin in the last week.

The guinea pigs were sacrificed 24 hours after the last dose of cisplatin. The oral extract was given per os after a 4-hour fasting period, once daily, starting one day before the first dose of cisplatin.

BAEP and OAEDP testing were done before starting the medication and 24 hours after the last dose. Weight gain and loss was also recorded. Pre- and post-treatment results were compared.

### Statistical Method

The data for each ear of each guinea pig were analyzed separately as a single datum, after which data was grouped; the reason for this was that each ear had different responses in the test and there was inter-individual variation within normal limits, particularly with OAEDP testing.

The ANOVA test was used for analyzing BAEP test results; the significance level was 5%. The chi-square test was used for analyzing OAEDP test results; the significance level was also 5%.

## RESULTS

### OAEDP

Table 1 shows the means for DP differences before and after the treatment, including the statistical test results. The group treated only with the aqueous extract (group 2) showed statistically significant differences in all frequency values (p<0.001) compared to the group treated only with cisplatin (group 1). We found that there were statistical differences between the groups treated with the aqueous extract and cisplatin only at the geometric mean values of 7206, 5434 and 2730 (groups 3,4 and 5) compared to group 1 (only cisplatin); these differences were not found in the other five frequencies.

**Table 1:** Mean DP-NF values (pre- minus DP-NF) following treatment in each group according to geometric mean of frequencies (geometric mean).

				Frequencies (geometric mean)				
Groups	7206	5434	3616	2730	1818	1352	886	676
1	37,33	35	15,5	23	18	4	7,67	7
2	-11.6	-2.1	-1.5	-4.6	-0.9	-1.4	4.2	-1.2
3	52,0	40,0	31,0	21,3	16,3	19,3	1,5	10,0
4	28,0	21,6	22,3	16,0	21,0	4,0	24,0	6,7
5	18	23,10	20,5	28	21	6,67	14	7,7
p	< 0.001	0.018	0.14	0.001	0.694	0.40	0.572	0.230

### BAEP

Table 2 shows the pre- and post-medication test results. The mean (M) and the standard deviation (SD) are shown.

**Table 2:** Mean value and SD of PTE before and after the treatment (M - mean; SD - standard deviation).

Groups					
	1	2 *	3**	4**	5**
Mean	90	42	98	89	91
SD	16,8	7	14	11	15
n	18	10	20	10	10

* p < 0.001 (group 1 × group 2)

** p= 0.31 (group 1 × groups 3, 4 and 5)

Group 1 showed a statistically significant threshold increase (p<0.001) following medication compared to the group that was given only the aqueous extract (group 2). There were no statistically significant differences between the responses of groups 3,4 and 5 (groups given cisplatin and the aqueous extract) and that of group 1 (only cisplatin) (p=0.31).

### Scanning electron microscopy

**Figure 1:**
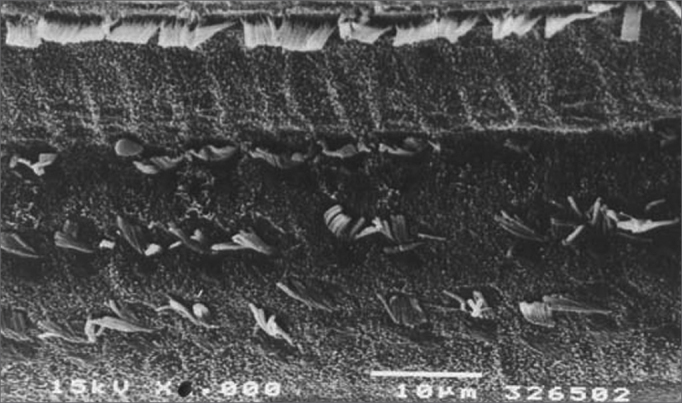
Scanning electron microscopy of a group 1 animal. Note extensive OHC injury in the basal portion of the cochlea. Scale = 10*µ*M; 1500X magnification.

The morphological investigation showed that there was severe injury (over 70% loss of OHCs) and complete (100%) loss of OHCs observed by scanning electron microscopy in some guinea pigs in the middle (and mostly) the basal gyri of guinea pigs treated only with cisplatin (Figure 1). Guinea pigs treated with the aqueous extract, regardless of the dose, showed severe injuries, but with preservation of some OHCs.

**Figure 2:**
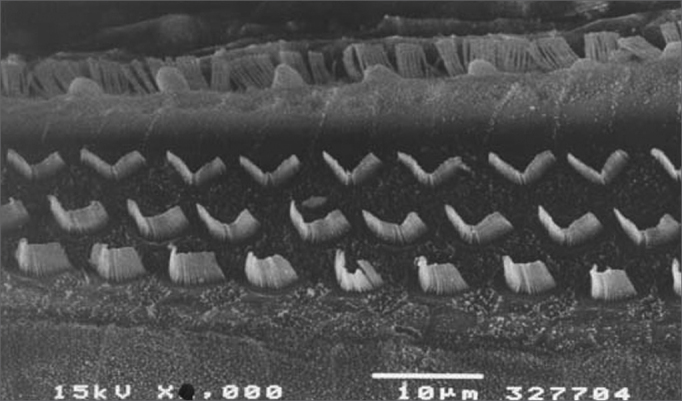
Scanning electron microscopy of a group 2 animal. Note preserved cilia in OHCs in the basal portion of the cochlea. Scale = 10*µ*M; 2000X magnification.

Figure 1 shows an EM image of a group 1 guinea pig in which extensive OHC injury is seen in the basal gyrus. Figure 2 shows an EM image of OHCs from group 2 guinea pigs in which anatomical structures are preserved.

**Figure 3:**
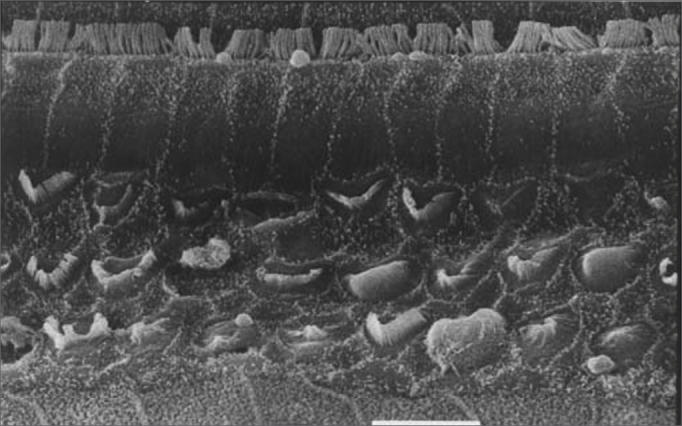
Scanning electron microscopy of a group 3 animal (EA and cisplatin treated). Note extensive OHC injury in the basal portion of the cochlea. Scale = 10*µ*M; 2000X magnification.

**Figure 4:**
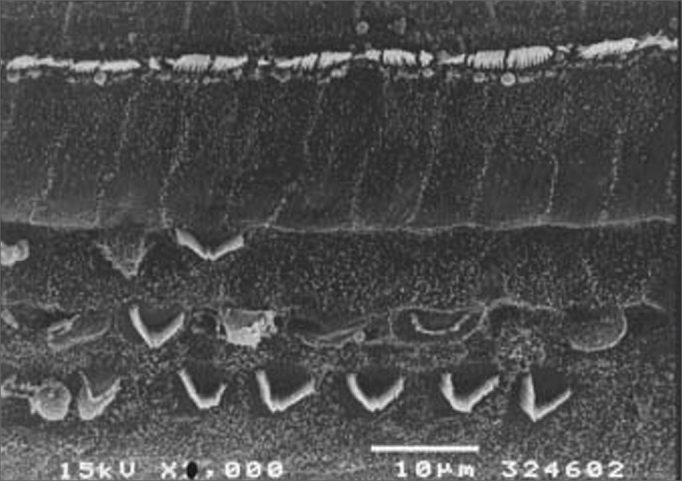
Scanning electron microscopy of a group 4 animal (EA and cisplatin treated - 3g/d). Note less intense OHC injury in the basal portion of the cochlea. Scale = 10*µ*M; 2000X magnification. Maytenus ilicifolia in the prevention of cisplatin-induced ototoxicity.

**Figure 5:**
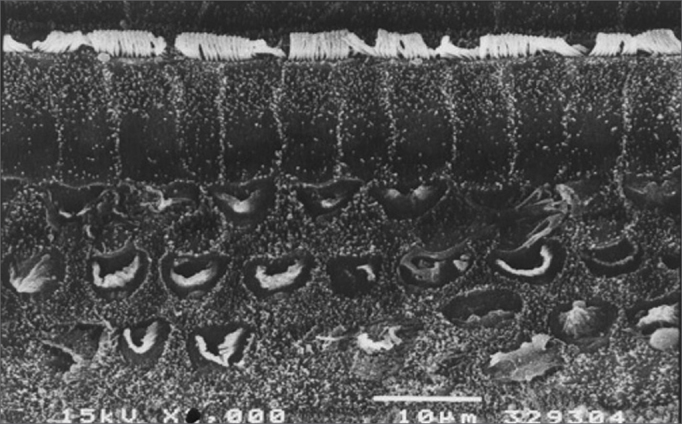
Scanning electron microscopy of a group 5 animal (EA and cisplatin treated during 3 weeks). Note extensive OHC injury in the basal portion of the cochlea. Scale = 10*µ*M; 2000X magnification.

Figures 3, 4 and 5 are images of basal gyri OHCs from groups 3, 4 and 5 guinea pigs, showing large areas in which hair cells are absent and preservation of some cells and anatomical structures.

### Clinical Status and Weight

The fur of guinea pigs treated jointly with the aqueous extract was not bristled, their food and water intake was not decreased, they were more active and the physical aspect was good. Exceptions were guinea pigs treated for three weeks (Table 3).

**Table 3:** Variation of the mean weight of guinea pigs during the treatment and on the day of euthanasia, and mean loss (-) or gain (+).

Groups	Mean - 1st day (weight g) )	Mean - 5th day (weight g)	Mean - 6th day (weight g)	Day of euthanasia (weight g)	Variation (weight g)
1	497.5	469	457.4	427.2	-70.2
2	470.4	486	480.8	49.06	20.2
3	400.5	370	357.5	347.9	-52,6
4	426	411	408	388.6	-37.4
5	531.6	482.8	481.8	456.8	-74.8

The mortality of the cisplatin only group (group 1) was 50%; there was no mortality in groups 2, 4 and 5. In group 3, 20% of animals did not survive more than 5 days.

## DISCUSSION

It is estimated that the Brazilian flora consists of between 40,000 and 60,000 plant species; it is thus a country with a major potential for producing medications. Maytenus ilicifolia has been widely studies, and its medicinal effects have been well demonstrated.^20^, ^21^, ^22^, ^23^, ^24^

Mattei and Carlini11 found that the extract of Maytenus ilicifolia has a significant in vitro antioxidant effect; it inhibits the lipoperoxidation process in rat brains by producing reactive species (antioxidant agents).

Oliveira et al.^22^ studied plasma red blood cells and proteins using radioactive technetium and found that there appeared to be antioxidant agents in the extract of this plant.

Melo et al.^23^ induced free radical production in E. Coli type bacteria previously treated with SnCl2 and compared the antioxidant properties of Maytenus ilicifolia, Cymbopogon citratus, and Baccharis genistelloides. Maytenus had the best effect of the three plants.

In our study, the aqueous extract of Maytenus ilicifloia was given in 1g/d doses during 8 days and 3g/d during 8 days, 24 hours before and jointly with cisplatin, to verify a possible otoprotective action of this plant due to its antioxidant mechanism.

The aqueous extract of Maytenus ilicifloia did not block cisplatin-induced OHC injury, regardless of the dose, as confirmed by auditory testing. Although the results of OAEDP testing showed statistically significant responses at some frequencies compared to the cisplatin group, there were poor responses at most frequencies. This may be explained by observing the scanning EM of some guinea pigs in which most of the OHCs were injured, but some rows of these cells were still preserved or had structural changes with no loss of cilia, suggesting that there may be favorable responses at certain OAEDP frequencies.

The aqueous extract also preserved the physical status of the animals; these animals had less weight loss and lower lethality. The aqueous extract as an antioxidant-producing drug used prior to cisplatin for otoprotection was not effective for preserving the hearing of guinea pigs (group 5).

There are some hypotheses for explaining our results. The aqueous extract of Maytenus ilicifolia appears to preserve the clinical status of guinea pigs exposed to cisplatin, possibly by an antioxidant action on other organs, as weight loss and mortality was clearly different among the groups. The metabolism in these organs, however, is different from that in the auditory apparatus; the aqueous extract of Maytenus ilicifolia was unable to block fully the ototoxic action of cisplatin in auditory organs specifically. A second hypothesis is that the aqueous extract of Maytenus ilicifolia did not cross the blood-brain barrier, and thus did not reach the cochlea; this would explain its effect in some organs only. A third hypothesis is that the free radical blocking mechanism by the aqueous extract of Maytenus ilicifolia was not sufficient to avoid cisplatin injury of the Corti organ at the doses used in this study.

## CONCLUSION

The aqueous extract of Maytenus ilicifolia did not have a sufficient otoprotective effect against cisplatin in this study; its use does foster improved physical conditions in the animals and decreases the lethality of cisplatin.
